# Anterior Segment OCT Features of Cytomegalovirus Endotheliitis Mimicking Graft Rejection After DSAEK: Report of Two Cases and Diagnostic Implications

**DOI:** 10.1155/crop/2019761

**Published:** 2026-07-27

**Authors:** Giovanni Rubegni, Alex Malandrini, Giorgia Guidetti, Giulia Spadavecchia, Gian Marco Tosi

**Affiliations:** ^1^ Ophthalmology Unit, Department of Medicine, Surgery and Neurosciences, University of Siena, Siena, Italy, unisi.it

**Keywords:** anterior segment OCT, cytomegalovirus endotheliitis, DSAEK, endothelial keratoplasty, graft rejection, viral endotheliitis

## Abstract

**Purpose:**

The purpose of the study is to describe anterior segment optical coherence tomography (AS‐OCT) findings in cytomegalovirus (CMV) endotheliitis occurring after Descemet stripping automated endothelial keratoplasty (DSAEK) and to emphasize their role in the differential diagnosis between viral endotheliitis and immune‐mediated graft rejection.

**Methods:**

Two cases of CMV‐associated endotheliitis following uneventful DSAEK were retrospectively analyzed. Clinical presentation, intraocular pressure changes, AS‐OCT features, virological assessment by aqueous humor polymerase chain reaction (PCR), treatment, and outcomes were evaluated.

**Results:**

Both patients developed postoperative graft dysfunction characterized by corneal edema, Descemet membrane folds, and elevated intraocular pressure, initially suggestive of acute allograft rejection. AS‐OCT consistently demonstrated a characteristic pattern consisting of graft folds associated with hyperreflective endothelial precipitates, even when slit‐lamp examination was limited by severe corneal edema. Aqueous humor PCR testing confirmed CMV DNA in both cases. Targeted antiviral therapy with oral valganciclovir led to resolution of endothelial inflammation and improvement of graft morphology. One patient required repeat endothelial keratoplasty due to irreversible endothelial damage despite virological control.

**Conclusions:**

CMV endotheliitis should be considered in cases of atypical graft dysfunction after DSAEK, particularly when associated with elevated intraocular pressure and suboptimal response to corticosteroid therapy. AS‐OCT may represent a valuable adjunctive tool in the immunological assessment of endothelial keratoplasty by supporting early recognition of viral endotheliitis and guiding appropriate antiviral management.

## 1. Introduction

Descemet stripping automated endothelial keratoplasty (DSAEK) is a selective and effective treatment for the management of corneal endothelial dysfunction, including Fuchs′ dystrophy and pseudophakic bullous keratopathy [1–3]. Despite excellent visual outcomes, postoperative complications such as graft detachment, rejection, interface fluid accumulation, and infectious endotheliitis remain clinically relevant [4, 5].

Cytomegalovirus (CMV) endotheliitis has emerged as an underrecognized cause of graft dysfunction following endothelial keratoplasty, particularly in immunocompetent patients [6, 7]. Its clinical presentation often overlaps with acute allograft rejection, sharing features such as corneal edema, graft folds, keratic precipitates (KPs), and elevated intraocular pressure (IOP), frequently leading to diagnostic delay and inappropriate escalation of corticosteroid therapy [8]. Differentiation between these entities is critical, as the management differs significantly [9, 10].

Anterior segment optical coherence tomography (AS‐OCT) provides high‐resolution noninvasive imaging of the cornea layers and graft‐host interface, and it is increasingly used in the postoperative assessment of endothelial keratoplasty [11, 12]. However, specific AS‐OCT features suggestive of viral endotheliitis, and particularly CMV infection, remain insufficiently characterized.

We report two cases of CMV endotheliitis following DSAEK, highlighting an AS‐OCT pattern characterized by graft folds associated with hyperreflective precipitates, which contributed to early diagnostic suspicion and timely antiviral treatment.

## 2. Case Report

All surgical procedures were performed by the same experienced corneal surgeon (A.M.). Written informed consent was obtained from both patients, and the study adhered to the tenets of the Declaration of Helsinki.

### 2.1. Case 1

A 54‐year‐old male with a past medical history of hypertension and hypercholesterolemia was referred to our Cornea Clinic for progressive visual deterioration in the right eye, attributed to bilateral Fuchs′ endothelial dystrophy. At presentation, best corrected visual acuity (BCVA) in the affected eye was 20/200. Slit‐lamp examination confirmed characteristic signs of Fuchs′ dystrophy and nuclear lens opacity. IOP was 17 mmHg, and dilated fundus examination was normal. The patient was using topical loteprednol 0.5% six times daily and topical hypertonic saline 5% four times daily in the right eye. The left eye showed early signs of Fuchs′ dystrophy but was otherwise unremarkable.

The patient underwent an uncomplicated combined procedure involving phacoemulsification with intraocular lens (IOL) implantation and DSAEK. Early postoperative follow‐ups revealed a well‐positioned, adherent graft with resolution of corneal edema and central corneal thickness (CCT) measured 594 *μ*m.

Three months postoperatively, the patient presented to the ophthalmic emergency department reporting acute visual loss in the operated eye; BCVA was 20/200. Slit‐lamp examination revealed multiple epithelial bullae with a central epithelial defect, pronounced stromal edema, and an edematous endothelial graft with deep folds that nevertheless remained adherent. IOP was elevated at 29 mmHg. AS‐OCT (MS‐39, Costruzione Strumenti Oftalmici, Florence, Italy) showed a markedly thickened graft with deep folds but no KPs initially: CCT increased to 829 *μ*m.

**Figure 1:**
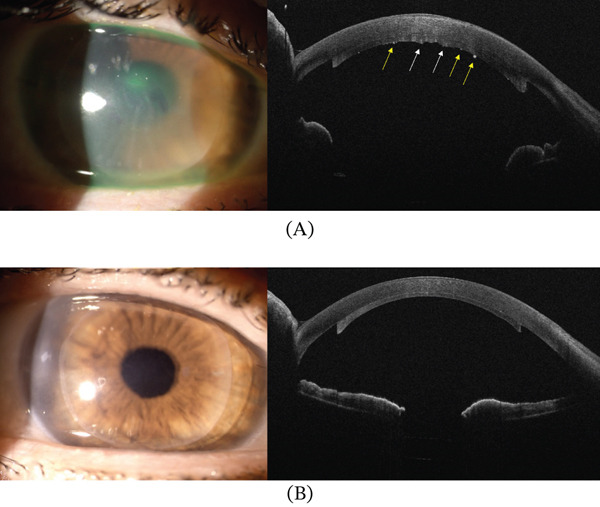
(A) Left: slit‐lamp photograph showing diffuse corneal edema, a central epithelial defect, and a deep graft fold with keratic precipitates. Right: AS‐OCT image demonstrating marked graft thickening with deep Descemet membrane folds (white arrows) and hyperreflective keratic precipitates (yellow arrows). (B) Left: slit‐lamp photograph showing clinical clearing of the cornea with resolution of stromal edema. Right: AS‐OCT image showing reduced graft thickness with resolution of Descemet membrane folds and keratic precipitates.

Presuming acute graft rejection, high‐dose topical corticosteroids were initiated (1.5 mg/mL dexamethasone phosphate six times daily), along with IOP‐lowering therapy consisting of fixed‐combination timolol/dorzolamide and brimonidine (both twice daily) and a single subconjunctival injection of dexamethasone (8 mg/2 mL). After 3 days of treatment, corneal edema persisted, preventing adequate visualization of corneal details on slit‐lamp examination: Repeat AS‐OCT demonstrated persistent graft folds and newly visible hyperreflective KPs located at their apex (Figure 1A).

Given the atypical response to corticosteroids, elevated IOP, and the delayed appearance of KPs as revealed on AS‐OCT, viral endotheliitis was suspected, and empirical antiviral therapy was initiated while awaiting aqueous humor polymerase chain reaction (PCR) results. Oral acyclovir (800 mg five times daily) was commenced, and an aqueous humor sample was obtained for PCR testing. Within days, there was minimal clinical improvement. PCR analysis returned positive for CMV DNA. Consequently, acyclovir was discontinued, and oral valganciclovir 900 mg twice daily was initiated.

In the following weeks, the patient showed gradual improvement with reduction in corneal edema, flattening of graft folds, and resolution of KPs (Figure 1B). Subsequently, corticosteroid therapy was tapered, and IOP‐lowering medications were discontinued.

After 4 months of continuous oral antiviral therapy and sustained clinical stability, the patient′s visual acuity improved to 20/25: CCT decreased to 567 *μ*m, consistent with restoration of corneal transparency. Valganciclovir was continued at a dosage of 900 mg twice daily for 6 weeks, then reduced to 900 mg once daily for an additional 6 weeks, after which the patient was transitioned to maintenance therapy with topical ganciclovir 0.15% gel once daily. During this period, the patient′s complete blood count and serum creatinine levels were monitored. At the 12‐month follow‐up, the patient remained clinically stable, with a clear cornea, no recurrence of inflammation or edema, and a BCVA of 20/25.

### 2.2. Case 2

A 73‐year‐old woman with a medical history of hypertension and polymyalgia rheumatica presented with long‐standing corneal edema in the right eye secondary to pseudophakic bullous keratopathy. Cataract surgery had been performed 3 years earlier, complicated by cystoid macular edema, which was partially responsive to two intravitreal injections of nonsteroidal anti‐inflammatory agents (triamcinolone acetonide). At the time of presentation, BCVA in the right eye was 20/200, and IOP was 15 mmHg. Topical treatment in the affected eye included 5% sodium chloride drops, applied three times daily.

Slit‐lamp examination revealed diffuse stromal edema, epithelial microbullae, inferior paracentral epithelial fibrosis, and a stable posterior chamber IOL. Fundus examination showed an epiretinal membrane. AS‐OCT demonstrated increased corneal thickness with prominent endothelial folds and subepithelial bullae; CCT measured 635 *μ*m. Examination of the fellow (left) eye revealed no significant abnormalities in either the anterior or posterior segment.

The patient underwent uncomplicated DSAEK 1 month later. On postoperative day 3, although the graft was fully attached, the cornea appeared edematous. BCVA was 20/200, and IOP was 22 mmHg. During the acute phase, AS‐OCT confirmed increased corneal thickness measured 803 *μ*m but did not show KPs at that time. A presumptive diagnosis of graft rejection was made, prompting the initiation of high‐dose topical corticosteroid therapy with 1.5 mg/mL dexamethasone phosphate administered six times daily, along with a single subconjunctival injection of dexamethasone (8 mg/2 mL).

**Figure 2:**
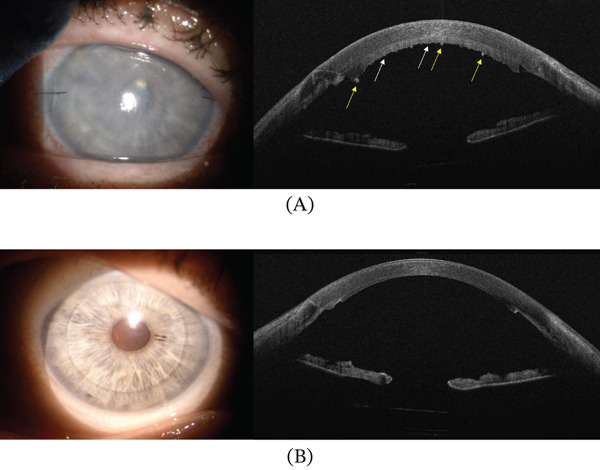
(A) Left: slit‐lamp photograph showing severe corneal edema with epithelial microbullae and impaired visualization of the anterior chamber. Right: AS‐OCT image demonstrating increased corneal thickness with graft folds (white arrows) and hyperreflective keratic precipitates (yellow arrows). (B) Left: slit‐lamp photograph showing a clear cornea with a fully attached graft. Right: AS‐OCT image demonstrating normalized corneal architecture with resolution of graft folds and absence of keratic precipitates.

By postoperative day 7, the cornea remained edematous, and visualization of the anterior chamber was severely impaired. Right eye visual acuity further declined to counting fingers. AS‐OCT revealed distinct hyperreflective endothelial precipitates located at the apex of the graft folds (Figure **2**A). IOP was elevated at 28 mmHg. Aqueous humor sampling and subsequent PCR analysis confirmed CMV DNA positivity.

Valganciclovir 900 mg twice daily was promptly initiated, and topical corticosteroid therapy (dexamethasone phosphate 1.5 mg/mL six times daily) was continued in combination with topical IOP‐lowering therapy (fixed‐combination timolol/dorzolamide twice daily). Within 4 weeks, KPs resolved, and graft folds diminished substantially. BCVA improved to 20/100, and IOP normalized to 18 mmHg. However, despite virological control and appropriate medical therapy, central stromal edema persisted, and CCT remained increased at 770 *μ*m. Given the persistence of poor visual acuity and stromal edema despite graft attachment, repeat DSAEK under systemic antiviral coverage was considered appropriate, in line with previous reports of CMV‐related endothelial failure [8, 10]. The second surgery was uneventful.

One month after the reintervention, the graft demonstrated proper adherence with CCT decreasing to 540 *μ*m, consistent with restoration of corneal transparency, and BCVA improving to 20/40. (Figure **2**B).

The patient remained on valganciclovir at 900 mg twice daily for the initial 6 weeks, followed by a step‐down to 900 mg once daily for another 6 weeks. Thereafter, given the patient′s good clinical response, systemic therapy was discontinued and replaced with topical maintenance using ganciclovir 0.15% gel applied once daily.

At 6‐month follow‐up, clinical status remained stable with no recurrence of edema or inflammation.

## 3. Discussion

Endothelial cell loss remains the primary cause of graft failure after DSAEK. However, additional factors may significantly contribute to graft dysfunction, including interface fibrosis, inadvertent retention of Descemet′s membrane, eccentric trephination, epithelial ingrowth, allograft rejection, and infectious endotheliitis [13]. Among infectious causes, CMV endotheliitis is increasingly recognized as an important and often underdiagnosed entity following endothelial keratoplasty.

CMV endotheliitis is frequently misdiagnosed as immune‐mediated allograft rejection due to substantial overlap in clinical presentation. Both conditions may manifest with reduced graft transparency, Descemet membrane folds, and corneal edema, sometimes appearing early after surgery. This diagnostic ambiguity is clinically relevant, as the management strategies differ substantially: rejection requires intensification of immunosuppression, whereas CMV endotheliitis mandates prompt antiviral therapy, and excessive corticosteroid use may exacerbate viral replication and endothelial damage.

Viral endotheliitis following endothelial keratoplasty represents a heterogeneous spectrum, primarily involving herpes simplex virus (HSV) and CMV. HSV endotheliitis more commonly reflects recipient viral reactivation and may present with linear or focal endothelial lesions, occasionally mimicking fungal interface infection, particularly after Descemet membrane endothelial keratoplasty (DMEK) [14]. In contrast, CMV endotheliitis is more frequently associated with elevated IOP, pigmented and often linear KPs, coin‐shaped or nummular endothelial lesions involving both donor and recipient cornea, and disproportionate endothelial cell loss.

In our cases, the combination of increased IOP, delayed appearance of endothelial precipitates, and an AS‐OCT pattern characterized by graft folds associated with hyperreflective endothelial deposits at their apex raised suspicion of a viral etiology, particularly CMV endotheliitis, and prompted further virological investigation. This distinction is supported by epidemiological data showing that HSV‐1 DNA is detected in only a small proportion of failed DSAEK grafts, whereas CMV, although less common, has been consistently reported as a cause of post‐DSAEK endotheliitis.^7.^

Clinically, allograft rejection and CMV endotheliitis may closely resemble one another, particularly in the early postoperative period. However, elevated IOP and the presence of endothelial KPs, especially when pigmented, linear, or associated with coin‐shaped lesions, are more strongly suggestive of CMV endotheliitis [15, 16]. In the early stages, these findings may be subtle or masked by severe corneal edema, limiting slit‐lamp evaluation.

AS‐OCT is increasingly recognized as a valuable tool in both preoperative and postoperative assessment of endothelial keratoplasty. Previous studies have characterized KPs using AS‐OCT and demonstrated the utility of high‐definition OCT in predicting allograft rejection by measuring progressive thickening of the endothelium–Descemet membrane complex in high‐risk corneal transplants [17, 18]. In our cases, AS‐OCT consistently demonstrated a tomographic pattern characterized by graft folds associated with hyperreflective endothelial precipitates. This finding proved particularly helpful in raising suspicion of viral endotheliitis when severe corneal edema significantly limited slit‐lamp examination. In addition, the marked corneal opacity during the acute phase precluded reliable specular microscopy assessment, further highlighting the complementary diagnostic value of AS‐OCT in this clinical setting. Although not pathognomonic, this “fold–precipitates tomographic pattern” may represent an early imaging biomarker of viral endothelial inflammation, particularly CMV‐related disease. Although the underlying mechanism remains speculative, preferential accumulation of KPs at the apex of graft folds may reflect localized alterations in aqueous humor microdynamics or focal areas of increased endothelial mechanical stress. These microenvironmental changes could facilitate inflammatory cell adhesion and deposition at these sites. Our observations support the growing role of AS‐OCT as an adjunctive diagnostic tool in the postoperative evaluation of endothelial keratoplasty. Further prospective studies are warranted to determine the sensitivity and specificity of these AS‐OCT findings in distinguishing viral endotheliitis from immune‐mediated graft rejection.

## 4. Conclusion

CMV endotheliitis should be considered in cases of early or atypical graft dysfunction following DSAEK, particularly when associated with elevated IOP and poor response to corticosteroids. AS‐OCT may reveal a characteristic pattern of graft folds associated with hyperreflective endothelial precipitates, particularly in cases where the anterior chamber is difficult to explore, which can raise early suspicion of viral endotheliitis and prompt appropriate virological testing. Early recognition and targeted antiviral therapy are crucial to prevent irreversible endothelial damage and graft failure.

## Funding

No funding was received for this manuscript.

## Ethics Statement

Ethics committee approval was not required for this case report. Written informed consent for publication of clinical data and images was obtained from all patients.

## Consent

Written informed consent was obtained from both patients for the publication of this case report and any accompanying images.

## Conflicts of Interest

The authors declare no conflicts of interest.

## Data Availability

No datasets were generated or analyzed during the current study. Data sharing is not applicable to this article.
